# The Practical Medicine Series of Year Books

**Published:** 1907-04

**Authors:** 


**The Practical Medicine Series of Year Books.** Ten volumes. Series of 1906. Issued under the general editorial charge of **Gustavus P. Head,** M.D., Professor of Laryngology and Rhinology in the Chicago Post-Graduate Medical School. Vol. VIII. Materia Medica and Therapeutics; Preventive Medicine; Climatology and Forensic Medicine. Edited by **George F. Butler, Henry B. Favill, Norman Bridge, Daniel R. Brower** and **Harold N. Moyer.** Chicago: The Year Book Publishers. (Price, $1.25; entire series, $10.00).

This is one of a series of ten volumes designed to cover the entire field of medicine for a year and while the series is intended as a broad fund of general information for the general practitioner, that he may keep abreast of the progress of the world of medicine, its division into a series of separated volumes on special subjects of allied interest, makes it possible for specialists and those interested in special work to secure only those volumes which appeal to them. The eighth volume, now under consideration, deals with materia medica and therapeutics by George F. Butler, professor of medicine in the Post-Graduate medical school, Chicago; preventive medicine and climatology, by Henry B. Favill, professor of therapeutics and preventive medicine in Rush Medical College; forensic medicine and suggestive therapeutics, by Harold N. Moyer, professor of nervous and mental diseases in Rush Medical College. The book completely fills the purpose for which it is written and becomes one of a very valuable series which in their entirety comprise a resume of the entire field of medicine.

